# Effect of tobacco smoke exposure during pregnancy and preschool age on growth from birth to adolescence: a cohort study

**DOI:** 10.1186/1471-2431-14-99

**Published:** 2014-04-10

**Authors:** Ana Paula Muraro, Regina Maria Veras Gonçalves-Silva, Naiara Ferraz Moreira, Márcia Gonçalves Ferreira, André Luis Nunes-Freitas, Yael Abreu-Villaça, Rosely Sichieri

**Affiliations:** 1 Instituto de Saúde Coletiva, Universidade Federal de Mato Grosso, Cuiabá, Brazil; 2 Departamento de Alimentação e Nutrição, Universidade Federal do Mato Grosso, Cuiabá, Brazil; 3 Departamento de Nutrição Social e Aplicada, Instituto de Nutrição Josué de Castro, Universidade Federal do Rio de Janeiro, Rio de Janeiro, Brazil; 4 Laboratorio de Neurofisiologia, Departamento de Ciências Fisiológicas, Instituto de Biologia Roberto Alcântara Gomes, Universidade do Estado do Rio de Janeiro, Rio de Janeiro, Brazil; 5 Departmento de Epidemiologia, Instituto de Medicina Social, Universidade do Estado do Rio de Janeiro, Rio de Janeiro, Brazil

**Keywords:** Smoking, Growth, Body height, Adolescent, Longitudinal studies

## Abstract

**Background:**

There is strong evidence of an association between maternal smoking during pregnancy and restriction of intrauterine growth, but the effects of this exposure on postnatal linear growth are not well defined. Furthermore, few studies have investigated the role of tobacco smoke exposure also after pregnancy on linear growth until adolescence. In this study we investigated the effect of maternal smoking exposure during pregnancy and preschool age on linear growth from birth to adolescence.

**Methods:**

We evaluated a cohort of children born between 1994 and 1999 in Cuiabá, Brazil, who attended primary health clinics for vaccination between the years 1999 and 2000 (at preschool age) and followed-up after approximately ten years. Individuals were located in public and private schools throughout the country using the national school census. Height/length was measured, and length at birth was collected at maternity departments. Stature in childhood and adolescence was assessed using the height-for-age index sex-specific expressed as z-score from curves published by the World Health Organization. Linear mixed effects models were used to estimate the association between exposure to maternal smoking, during pregnancy and preschool age, and height of children assessed at birth, preschool and school age, adjusted for age of the children.

**Results:**

We evaluated 2405 children in 1999–2000, length at birth was obtained from 2394 (99.5%), and 1716 at follow-up (71.4% of baseline), 50.7% of the adolescents were male. The z-score of height-for-age was lower among adolescents exposed to maternal smoking both during pregnancy and childhood (p < 0.01). Adjusting for age, sex, maternal height, maternal schooling, socioeconomic position at preschool age, and breastfeeding, children exposed to maternal smoking both during pregnancy and preschool age showed persistent lower height-for-age since birth to adolescence (coefficient: −0.32, p < 0.001) compared to non-exposed. Paternal smoking at preschool age was not associated with growth after adjustment for confounders.

**Conclusion:**

Exposure to maternal smoking not only during pregnancy, but also at early childhood, showed long-term negative effect on height of children until adolescence.

## Background

Growth failure in early life is a strong determinant of final adult height in low and middle-income country [1,2]. Short stature is associated with adverse functional consequences, including in cognition and educational performance, reduced adult income, lost productivity and, when accompanied by excessive weight gain later in childhood, increased risk of nutrition-related chronic diseases [3]. It is known that linear growth is influenced by genetic and environmental factors [4], among the latter, exposure to smoking during pregnancy or childhood could affect growth.

There is strong evidence of an association between smoking during pregnancy and low birth weight and restriction of intrauterine growth [5], but the effects of this exposure on postnatal linear growth are not well defined. Studies have shown that exposure to tobacco during pregnancy elicits persistent effects on height during childhood [6-9]. Recently, Howe and colleagues [10] observed that height deficits for offspring of women who smoked during pregnancy persisted into childhood, in a large prospective birth cohort study in South-West England. A dose–response association has also been observed with linear growth reduction in children, which depends on the amount of maternal smoking during pregnancy [7,8,11]. Other studies, however, do not support the finding of long-term effects of prenatal exposure to tobacco on postnatal height [12-14].

Few studies evaluated whether the effect of maternal smoking during pregnancy on linear growth at childhood persisted until adolescence. Gigante et al. [15] showed that 19 year-old Brazilian girls exposed to maternal smoking during pregnancy had lower height than those who were not exposed, in analyses adjusted for potential confounders. In contrast, Heffner et al. [16], studying 18 years old adolescents, did not observe negative association between maternal smoking during pregnancy and adolescent’s height after adjustment for potentials confounders and birth weight. In addition, children exposed to prenatal smoking are more likely to be exposed to postnatal passive smoking [8], but few studies account for this period of exposition.

In a previous analysis of the cohort of the present study, evaluated at preschool age, maternal prenatal and postnatal smoking had a strong inverse association with height-for-age of the children, even after adjustment for variables related to the socioeconomic position of families [17]. The aim of the present analysis is to evaluate whether the exposure to maternal smoking during pregnancy and preschool age is associated with linear growth from birth to adolescence, approximately ten years after the first evaluation.

## Methods

A cohort of children born between 1994 and 1999 in Cuiabá, Brazil, who attended primary health clinics for vaccination in the period from May 1999 to January 2000 was evaluated. A full description of the sampling plan has been described previously [17]. Briefly, from the 38 vaccination clinics, ten were randomly selected, and the parents or guardians of approximately 240 children randomly selected at each clinic were interviewed (n = 2405). All guardians who were accompanying their children were invited to participate; the refusal rate was 0.4%. The coverage in Brazil for DPT vaccine (vaccine against diphtheria, whooping cough and tetanus) at that point in time was 97%.

This cohort has a mixed design with both non concurrent and concurrent follow-up components. Information about birth (length and weight) was obtained from hospitals records, but all outcomes and major expositions, when the children were from zero to five years old (preschool age) and when they were between 10 and 17 years, were measured or assessed through questionnaires by the researchers.

In Brazil, approximately 95% of children aged 10 to 14 years and 78% of children aged 15 to 17 years attend school [18]. The annual School Census in Brazil was used to follow-up the cohort. The national census is coordinated by the National Institute of Educational Studies Anísio Teixeira (INEP) and includes all public and private schools throughout the country. Through the child’s name, date of birth and name of the mother, 86.8% of the adolescents and their schools were identified. In addition, through the National Mortality Information System [19], five deaths were identified. We interviewed and examined 1716 (71.4% of 2405 evaluated at preschool age) adolescents at their schools between 2009 and 2011 corresponding to visiting all adolescents still living in Cuiabá and neighboring cities, those living in other 17 cities, and five other capital cities (Brasília, Goiânia, Rio de Janeiro, São Paulo and Campo Grande).

**Figure 1:**
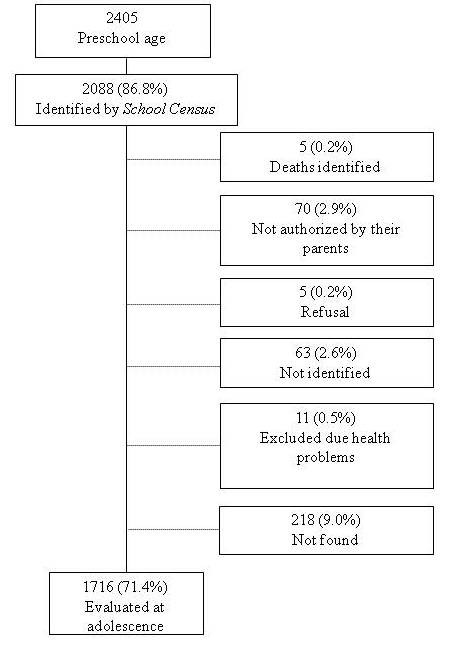
Flow chart of study population.

As shown in Figure 1, from all evaluated at preschool age (2405): 11 (0.4%) with incapacitating health problems were excluded from the interview, 70 (2.9%) adolescents were not authorized by their parents or guardians to participate in the survey, 63 (2.6%) did not come to the school on the three attempts to measure them, five (0.2%) adolescents refused to participate, and we were unable to evaluate 218 (9.0%) adolescents due, for example, to live in distant cities. Further details are described in Gonçalves-Silva et al. [20].

### Measures

Information about the child’s birth, sociodemographic characteristics of the families, breastfeeding and children’s exposure to passive smoking were obtained by an interview with the parents or guardians. Information on weight and length at birth was obtained directly from the child’s vaccination card or from the hospital record (most data came from the hospital record), and length was measured by the researchers using standard technique [21].

Height of the mothers was self-reported at first interview. Mothers were asked if they smoked during pregnancy and which trimester they smoked. Those who reported any amount of smoking in any trimester of pregnancy were classified as pregnancy smokers. Fathers or other member of the household who reported smoking at least one cigarette a day for at least one year were classified as smokers.

Paternal and maternal education was assessed at both study periods. Educational level was categorized into four groups: 0–4, 5–8, 9–11, and 12 years or more completed years of formal education.

Exposure to maternal smoking during pregnancy and early childhood was classified as no exposure (those who were not exposed during both periods), exposed only during pregnancy (those whose mothers reported having smoked during pregnancy but not during preschool age), exposed only during preschool age (when mothers reported not having smoked during pregnancy but smoked during preschool age of the children), and exposed to maternal smoking during both periods.

At school, adolescents were interviewed about smoking and socioeconomic factor using a pretested questionnaire; and anthropometric measurements were collected by trained field workers according to the techniques recommended by Lohman et al. [21].

To validate the responses regarding smoking among adolescents, the concentration of cotinine, the major metabolite of nicotine, was measured. Saliva samples were collected in a random sub-sample of 387 adolescents with the OraSure® oral sample collection device. Saliva was used because it is simple and non-invasive and is acceptable to this age group. The samples were analyzed by ELISA immunoassay (OraSure Technologies, Inc., Bethlehem, PA, USA) at the Laboratory of Neurophysiology in the Department of Physiological Sciences, University of the State of Rio de Janeiro. The minimum detectable concentration for cotinine was 3 ng/ml.

Owing to the low intensity of smoking in this age group, a cutoff of 5 ng/ml was chosen as a threshold for active tobacco use [22]. Values below 5 ng/ml were thus interpreted as no tobacco use in the preceding seven days or low level of exposure due to passive smoking only.

For analysis, the index of height-for-age and sex expressed in z-score according to the growth curves published by the World Health Organization (WHO) [23,24] was used. Scores were calculated using the WHO Anthro program, version 3.1. The cutoff for a deficit in height (stunting) was a z-score below −2 of the reference distribution, according recommended by WHO [25].

The socioeconomic position of families was based on the number of home appliances, cars, paid maids, and the educational level of the head of household, Brazilian Marketing Research Association criteria [26,27]. Birth weight was classified into the following four categories according to criteria of the WHO [25]: low birth weight (<2500 g), underweight (2500–2999 g), appropriate weight (3000–3999 g) and overweight (≥4000 g). Breastfeeding was classified in “any breastfeeding”, when mother reported that child has received breast milk with or without other drink, formula or other infant food.

### Data analysis

To determine biases associated with losses and censored data, we compared the baseline characteristics of participants and those lost to follow-up.

The mean z-score of height-for-age in childhood and adolescence according to demographic and socioeconomic characteristics, length and weight at birth, breastfeeding, maternal height, and exposure to passive smoking was compared using the Student’s t-test and analysis of variance (ANOVA).

Linear mixed effects models, using the procedure PROC MIXED in SAS software, were used to examine the effect of exposure to maternal smoking during pregnancy and childhood on height-for-age (in z-score) of the children over the three periods: at birth, preschool age (when children was zero to five years old), and at school (10 to 17 years old). Time in the models is the age of the child as a continuous variable (years) at each measurement. Models were tested for random effects (G matrix) of intercept and slope and both were included in the models. The structure chosen for G matrix was the unstructured type as suggested by Fitzmaurice et al. [28]. These models account for the correlation between repeated measurements and allow for incomplete outcome data [28]. To evaluate if there was a difference of linear growth rate over time between children exposed to maternal smoking during pregnancy and childhood in comparison with those who were not exposed, an interaction term of age of the child and maternal smoking was tested (age of the child *maternal smoking). The null hypothesis means that the difference of height-for-age between the groups is constant over time. Models were adjusted for all variables with p-value <0.20 at bivariate analyses, keeping in the analysis those changing the effect of maternal smoking exposure on growth. The final model is described by the formula:

The final model is described by the formula:

$$\begin{array}{ll} \;\text{BMI}\;z-{\text{score}}_{\text{it}}= & \beta 1+\beta 2*\text{Ageit}+\beta 3*\text{Maternal}\;\text{Smoking}1_{\text{it}}\\ & +\beta 4*\text{Maternal}\;\text{Smoking}2_{\text{it}}\\ & +\beta 5*\text{Maternal}\;\text{Smoking}3_{\text{it}}\\ & +\beta 7*{\text{Age}}_{\text{it}}*\text{Smoking}1_{\text{it}}\\ & +\beta 8*{\text{Age}}_{\text{it}}*\text{Smoking}2_{\text{it}}\\ & +\beta 9*{\text{Age}}_{\text{it}}*\text{Smoking}3_{\text{it}}+\;\beta 10*\text{Gender}\\ & +\beta 11*\text{Maternal}\;\text{Height}\\ & +\beta 12*\text{Economic}\;\text{class}+\beta 13*\text{Breastfeeding}\\ & +e_{\text{it}.} \end{array}$$

Where i represents individual, t represents time, β1-13 represent estimates, and e is error term.

Fitness of the models were examined graphically to assess normality of the residuals and satisfy regression requirements. Analyses were performed with Statistical Analysis Systems statistical software package, version 9.3 (SAS Institute, Cary, NC, USA).

The project was approved by the Ethics Committee of the Júlio Müller University Hospital, Federal University of Mato Grosso (651/CEP-HUJM/2009 Protocol). Parents or guardians of the participating adolescents signed a consent form.

## Results

Among 2405 children evaluated at childhood (1999/2000), length at birth was obtained from 2394 (99.5%), and 71.4% of them (n = 1716) were evaluated at adolescence (2009–2011), at ages between 10 and 17 years old. Only 5.3% of children and 1.2% of adolescents had low height-for-age. Loss to follow-up was greater among adolescents who had low height-for-age, mothers with less education and among those exposed to maternal smoking during pregnancy (Table 1).

**Table 1:** Sample size (N), characteristics of participants and follow-up rate

	**1999-2000**	**2009-2011**	**Follow-up rate**
**Age in years - mean and (SD)**	1.5 (1.4)	12.2 (1.5)	**-**
	**N (%)**	**N (%)**	**%**
**Age (in years)**			
<1	1186 (49.3)	842 (49.1)	71.0
1-2	512 (21.3)	370 (21.5)	72.3
>2	707 (29.4)	504 (29.4)	71.3
			*p* = 0.86
**Gender**			
Male	1224 (50.9)	870 (50.7)	71.1
Female	1181 (49.1)	846 (49.3)	71,6
			*p* = 0.76
**Birth weight (g)**			
≥ 4000	143 (6.9)	102 (5.9)	71.3
3000-3999	1619 (67.6)	1160 (67.6)	71.7
2500-2999	483 (20.1)	344 (20.1)	71.2
< 2500	160 (6.4)	110 (6.4)	68.7
			*p* = 0.89
Height-for-age at birth (z-score)^*^			
≥ −2 z-score	270 (11.1)	195 (11.3)	72.2
< −2 z-score	2123 (88.7)	1512 (62.9)	71.2
			*p* = 0.73
**BMI-for-age (z-score)**			
Thinness (< −2 z-score)	68 (2.8)	41 (2.4)	60.3
Adequate (≥ −2 to ≤ 1 z-score)	1857 (77.2)	1325 (77.2)	71.3
Overweight (>1 to ≤ 2 z-score)	371 (15.4)	270 (15.7)	72.8
Obesity (>2 z-score)	108 (4.5)	80 (4.7)	74.1
			*p* = 0.18
**Height-for-age (z-score)**			
≥ −2 z-score	146 (8.0)	90 (5.3)	61.6
< −2 z-score	2258 (93.9)	1626 (94.8)	72.0
			***p*** **= 0.01**
**Socioeconomic position**^**†**^			
A (high-income)	86 (3.6)	57 (3.3)	66.3
B	289 (12.0)	206 (12.0)	71.3
C	1019 (42.4)	743 (43.3)	72.9
D	807 (33.5)	577 (33.6)	71.5
E (low-income)	204 (8.5)	133 (7.7)	65.2
			*p* = 0.19
**Maternal schooling (years)**^**‡**^			
≥ 12	206 (8.6)	153 (8.9)	74.3
9 – 11	638 (26.5)	480 (28.0)	75.2
5 – 8	1363 (56.7)	956 (55.7)	70.1
0 – 4	177 (7.4)	113 (6.6)	63.8
			***p*** **= 0.02**
**Maternal smoking during pregnancy**			
Yes	271 (11.3)	167 (9.7)	61.6
No	2133 (88.7)	1549 (90.3)	72.6
			***p*** **< 0.01**

p value from Chi-square test; ^*^No information for 12 children.

^†^According to the criteria of the Brazilian Marketing Research Association (2003): based on the number of home appliances, cars and paid maids, and education level of the head of household.

^‡^In 1999, 21 mothers and 449 fathers didn’t live with their children.

Lower mean z-scores of height-for-age were found in older age groups, especially among adolescents aged 14 or over. Higher socioeconomic level, both at preschool age and at adolescence, and higher parental schooling was associated with higher average height-for-age, both during childhood and adolescence. In addition, children of mothers classified in higher tertiles of height and with greater birth weight showed higher mean z-score of height-for-age in both periods (Table 2).

**Table 2:** Mean and 95% Confidence Interval (95% CIs) of the height-for-age z-score, at preschool age (0 – 5 years old) and current (10 – 17 years old), of adolescents selected characteristics

	**N**	**Height-for-age 0 – 5 years**		**Height-for-age 10 – 17 years**	
		**Mean**	**95% CI**	**Mean**	**95% CI**
**Gender**					
Male	1224	−0.20	−0.28; −0.12	0.21	0.14; 0.28
Female	1181	−0.14	−0.22; −0.07	0.26	0.20; 0.33
		**p < 0.01**		p = 0.29	
**Age (years)**					
10	409	−0.24	−0.36; −0.13	0.27	0.17; 0.37
11	551	−0.11	−0.22; −0.01	0.31	0.22; 0.39
12	322	−0.16	−0.29; −0.04	0.31	0.19; 0.42
13	183	−0.12	−0.29; −0.05	0.25	0.10; 0.39
≥ 14	251	−0.26	−0.37; −0.15	−0.08	−0.19; 0.03
		p = 0.31		**p < 0.01**	
Socioeconomic position at preschool age^*^					
A (high-income)	86	0.31	0.03; 0.59	0.59	0.30; 0.87
B	289	0.13	−0.2; 0.28	0.41	0.29; 0.54
C	1019	−0.13	−0.21; −0.05	0.28	0.21; 0.35
D	807	−0.35	−0.45; −0.25	0.13	0.05; 0.21
E (low-income)	204	−0.33	−0.52; −0.15	−0.01	−0.17; 0.15
		**p < 0.01**		**p < 0.01**	
Current Socioeconomic position^*^					
A (high-income)	86	0.16	−0.05; 0.38	0.49	0.26; 0.71
B	603	0.00	−0.08; 0.09	0.33	0.25; 0.41
C	959	−0.29	−0.36; −0.21	0.17	0.11; 0.24
D e E (low-income)	68	−0.61	−0.92; −0.30	0.14	−0.40; 0.12
		**p < 0.01**		**p < 0.01**	
**Maternal schooling (years)**					
≥ 12	206	0.41	−0.09; 0.17	0.39	0.26; 0.51
9 – 11	638	−0.11	−0.18; −0.03	0.28	0.21; 0.35
5 – 8	1363	−0.31	−0.42; −0.20	0.15	0.06; 0.23
0 – 4	177	−0.44	−0.66; −0.23	−0.04	−0.25; 0.18
		**p < 0.01**		**p < 0.01**	
**Paternal schooling (years)**					
≥ 12	221	0.05	−0.09; 0.18	0.37	0.25; 0.49
9 – 11	555	−0.06	−0.14; 0.02	0.31	0.23; 0.38
5 – 8	1044	−0.03	−0.41; −0.19	0.17	0.08; 0.26
0 – 4	136	−0.27	−0.46; −0.07	0.20	0.03; 0.38
		**p < 0.01**		**p = 0.03**	
**Maternal height**					
1° tertile	795	−0.57	−0.66; 0.48	−0.14	−0.22; −0.66
2° tertile	795	−0.06	−0.15; 0.03	0.23	0.15; 0.31
3° tertile	794	0.11	0.01; 0.20	0.61	0.54; 0.69
		**p < 0.01**		**p < 0.01**	
**Birth weight (g)**					
≥ 4000	143	0.45	0.23; 0.66	0.55	0.38; 0.74
3000-3999	1619	−0.02	−0.08; 0.05	0.29	0.23; 0.35
2500-2999	483	−0.60	−0.69; −0.47	0.04	−0.06; 0.15
< 2500	160	−1.17	−1.40; −0.93	−0.06	−0.26; 0.14
		**p < 0.01**		**p < 0.01**	
**Breastfeeding**					
Any	1945	−0,24	−0.30; 0.19	0.24	0.18; 0.29
Never	460	0.09	−0.04; 0.22	0.22	0.10; 0.33
		p < 0.10		p = 0.72	
**Maternal smoking during pregnancy and childhood**					
During both periods	212	−0.56	−0.74; −0.38	−0,02	−0.21; 0.17
Only during childhood	76	−0.22	−0.48; 0.04	0,07	−0.19; 0.34
Only during pregnancy	59	−0.46	−0.76; 0.17	0,23	−0.19; 0.67
No smoking	2042	−0.14	−0.19; −0.09	0,24	0.22; 0.32
		**p = 0.01**		**p < 0.01**	

p value from *t* t*est* or ANOVA.

*According to the criteria of the Brazilian Marketing Research Association (childhood: 2003, adolescent: 2008): based on the number of home appliances, cars and paid maids, and education level of the head of household.

Missing values: current socioeconomic position: 2; maternal schooling: 21; paternal schooling: 449; maternal height: 4.

Most (80.2%) of mothers that smoked during pregnancy continue smoking at post-natal period (Table 2). Among mothers who smoked only during pregnancy (n = 59), 97.7% smoked only in the first trimester. The z-score of height-for-age was lower among adolescents exposed to maternal smoking both during pregnancy and during childhood compared with those who were never exposed (Table 2). Paternal smoking during childhood was associated with lower z-score of height-for-age only at preschool age, but when included at multivariable models not remained associated (p = 0.68) and did not affect the coefficient of association between maternal smoking and growth.

**Figure 2:**
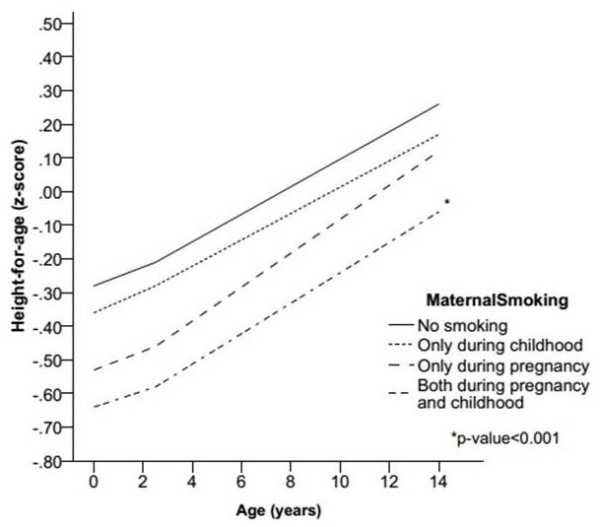
Predicted means of z-score of height-for-age from birth to adolescence, for exposure to maternal smoking during pregnancy adjusted for sex, maternal height, socioeconomic position at preschool age, and breastfeeding.

As shown in Figure 2, after adjusting for the confounding factors (sex, maternal height, socioeconomic position of family at pre-school age, and breastfeeding), exposure to maternal smoking both during pregnancy and preschool age conferred persistent negative effects on growth (Regression Coefficient = −0.32, p < 0.001).

The interaction term between time and categories of maternal smoking was not statistically significant (p = 0.71), indicating that there was no difference on the annual rate of growth among those who were exposed. Also, further adjustment for socioeconomic position at adolescence did not change substantially the associations. Lack of interaction may be due to the small sample size of two of the smoking categories and also to the limited number of follow-up measurements. The point estimates in Table 3, showed greatest effect for smoking at both periods since the regression coefficient for smoking only during pregnancy was −0.15, for smoking only during childhood was −0.05, and for both it was −0.33. The fact that only the p-value for smoking in both periods was statistically significant might be due to the small sample size.

**Table 3:** Regression coefficient of height-for-age (z-score) according linear mixed effect model

	**Coefficient**	**Standard error**	**p-value**
Maternal smoking			
During both periods	−0.33	0.077	<0.001
Only during preschool age	−0.05	0.100	0.67
Only during pregnancy	−0.20	0.140	0.15
No smoking	-	-	-
Time*Maternal smoking			
During both periods	0.004	0.008	0.57
Only during preschool age	−0.02	0.013	0.09
Only during pregnancy	0.01	0.016	0.52
No smoking	-	-	-
Age	0.04	0.002	<0.001
Gender			
Male	−0.06	0.035	0.05
Female	-	-	-
Maternal height	0.04	0.002	<0.001
Economic class	0.07	0.02	<0.001
Breastfeeding			
Yes	−0.06	0.04	0.18
No	-	-	-

Because most users of tobacco start smoking in early adolescence, active smoking could have had impaired growth; we included in the analyses smoking status of 65 (3.8%) of the 1716 adolescents who experimented tobacco. Adjustment for smoking status did not change the results since only 11 (0.6%) reported tobacco use in the 30 days preceding the survey (data not shown).

In the validation study in a sample of 387 adolescents, only 6 (1.5%) showed measurable cotinine concentrations; among those, only three (0.8%) had a concentration above the cutoff of 5 ng/ml [22].

## Discussion

The results of this study indicate that exposure to prenatal and postnatal maternal smoking had a persistent negative effect on height until adolescence; children who were exposed in these periods were shorter since birth until adolescence compared with those who were not exposed. Many studies had shown a negative effect of maternal smoking during pregnancy on height until childhood [6,8,10,11], but few have used individual growth analysis, which is an important approach to claify the association between maternal smoking early in life and childhood growth [29].

Analyses of birth cohort studies in Brazil showed that children of women who smoked during pregnancy had persistent lower height until 4 years [9] and also in adolescence [15]. In this Brazilian study, most of children exposed during pregnancy were exposed exclusively in first trimester.

Leary and collaborators [8] found a negative effect of maternal smoking during pregnancy in components of stature in offspring, and this effect was similar when the smoking data were analyzed separately for each trimester.

Howe et al. [30], using repeated measures from birth to 10 years old of an England birth cohort, suggested that children of smoking mothers grow more rapidly in infancy but more slowly later in childhood, but these differences were relatively small. Our study did not indicated statistically significant difference in annual growth rate from birth until preschool age and adolescence.

Socioeconomic position of the family is an important confounding in the association of tobacco exposure and growth. In Brazil, longitudinal studies have found a positive association between socioeconomic class and the height reached in late adolescence [16], and that socioeconomic background was a predictor of linear growth during the school-aged years [31]. Also, smoking prevalence is higher among lower-income families and individuals of low education [32]. The data of our cohort support this statement; there was a higher exposure to household smoking among families of lower socioeconomic position [33], but after adjusted analyses for socioeconomic level of the family at childhood and adolescence associations of maternal smoking with growth did not change substantially. In addition, the lack of association between paternal smoking during childhood and linear growth of the children is this analysis and other studies [8,9,11], also suggested that these results are not due familiar confounding factors.

During pregnancy, a hypothesis for the physiological mechanism of this association is the embryotoxic effects of nicotine or other toxic pollutants found in cigarette smoke that lead to delayed skeletal growth [34]. The stronger association of maternal smoking during childhood found in this study may be explained by the effect of smoking during the breastfeeding period and the fact that preschool-age children spend more time with their mothers and, therefore, are more susceptible to the harmful effects of tobacco smoke. The various toxic substances from tobacco, when present in breast milk, can inhibit growth by changing the supply and bioavailability of essential nutrients, such as zinc [35]. Furthermore, children exposed to maternal smoking have a greater risk of respiratory diseases than children whose father or any other resident of the household is a smoker [36,37], and it may be one possible mediator of impaired growth.

The prevalence of stunting at adolescence in the present study (1.2%) was low. A national study conducted by the Brazilian Institute of Geography and Statistics [38] between 2002 and 2003 showed a significant decrease in the prevalence of low height-for-age over the past decades. This decrease is probably due to the improved living and health conditions of the population that have been observed. In our sample a change in socioeconomic position was also observed between the two evaluations of the children. At first interview, approximately 40% were in classes D and E, but in the follow-up, ten years later, only 4% were in these classes.

Among the limitations of this study is the lack of information at preschool age regarding food consumption of the children, the adolescent puberty attainment, pre-pregnancy nutritional status, maternal alcohol or other drug use and the number of cigarettes smoked by the mother. Also, the rate of follow-up in this study was 72%, and selective loss was observed in this sample, with greater loss among children who showed low height-for-age and were exposed to tobacco smoke. This selective loss to follow-up may have biased the findings toward the null hypothesis.

The power of the study was also influenced by the low prevalence of maternal smoking compared to other countries, but this finding is consistent with others studies in Brazil, showing that smoking during pregnancy has declined substantially in the country over the last 20 years, during which time the country introduced many strong tobacco control policies [39].

As strengths of this study maternal height was assessed and its inclusion in the analysis helps, at least partially, to adjust for the effect of genetics on adolescent height [40]. On the other hand, information about maternal and paternal smoking was obtained by a questionnaire; thus, misclassification may have occurred, mainly about exposure during pregnancy that was retrospectively assessed. However, the self-reporting of this behavior appears to be an accurate measure. Cornelius and colleagues [13] measured environmental tobacco exposure of children through maternal report and a biological measure from the children (urinary cotinine level). The authors observed that the mother’s report of exposure captured a greater number of exposed children than the biological measure, and therefore, the information used in their analysis was the maternal report. All of these possible biases cause an underestimation of the impact of exposure on growth.

## Conclusion

In conclusion, maternal smoking during pregnancy and early childhood confers a long-term negative effect on height of children since birth to adolescence, emphasizing the importance of smoking cessation among women, not only during pregnancy.

## Competing interests

The authors declare that they have no competing interests.

## Authors’ contributions

The reported analysis included measurement of cotinine concentration due by the authors YAV and ALNF, specific longitudinal analysis due by APM, RS, and RMVGS, analysis of School Census due by APM and NFM, data collected at baseline and follow-up due by RMVGS, MGF, APM, and NFM. All authors read and approved the final manuscript.

## Pre-publication history

The pre-publication history for this paper can be accessed here:

http://www.biomedcentral.com/1471-2431/14/99/prepub
